# The Approach of Physiotherapists in the Management of Patients with Persistent Pain and Comorbid Anxiety/Depression: Are There Any Differences between Male and Female Professionals?

**DOI:** 10.3390/medicina60020292

**Published:** 2024-02-08

**Authors:** Michele Chiesa, Gregorio Nicolini, Massimiliano Buoli

**Affiliations:** 1 Faculty of Medicine, University of Milan, 20122 Milan, Italy; fisiomike@gmail.com; 2 Department of Mental Health, Department of Biomedical and Clinical Sciences Luigi Sacco, Luigi Sacco Hospital, University of Milan, Via G.B. Grassi, 74, 20157 Milan, Italy; gregorio.nicolini@unimi.it; 3 Department of Neurosciences and Mental Health, Fondazione IRCCS Ca’ Granda Ospedale Maggiore Policlinico, Via F. Sforza 35, 20122 Milan, Italy; 4 Department of Pathophysiology and Transplantation, University of Milan, 20122 Milan, Italy

**Keywords:** physiotherapy, depression, anxiety, gender, management

## Abstract

*Background and Objectives*: Chronic pain is a prevalent condition that is frequently complicated by mood and anxiety disorders. The purpose of the present article is to identify differences in the management of patients with chronic pain and anxiety/mood disorders depending on the physiotherapists’ gender. *Materials and Methods*: An ad hoc questionnaire was developed and sent to 327 physiotherapists by e-mail. The two groups identified by gender were compared by unpaired-sample *t* tests for continuous variables and χ^2^ tests for qualitative ones. A binary logistic regression was then performed with factors resulting as statistically significant in univariate analyses as independent variables and gender as a dependent one. *Results*: Female physiotherapists exhibited a higher level of confidence than male physiotherapists in administering continued physiotherapy for patients affected by Generalized Anxiety Disorder (GAD) (*p* = 0.01), as well as for individuals who had previously engaged with a mental health professional (*p* = 0.01). Furthermore, female physiotherapists believed that pharmacotherapy was less associated with motor side effects (*p* < 0.01) and more frequently recognized the importance of training to identify affective disorders (*p* = 0.01) and the need for more education in mental health (*p* = 0.01). The binary logistic regression model confirmed that female professionals were less likely to work = freelance (*p* = 0.015) and were more confident in the receival of physiotherapy by patients with GAD (*p* = 0.05). *Conclusions*: Female physiotherapists compared to male ones seem to be more comfortable with patients affected by mental conditions and to be more aware of the need for training on mental health. Further studies are needed to confirm the results of the present study.

## 1. Introduction

Chronic pain is a common condition in the global population [1] and it is often concomitant with psychiatric comorbidities, such as anxiety and depressive disorders [2]. The co-occurrence of chronic pain and psychiatric disorders is responsible for high disability and poor quality of life [3]. The healthcare costs associated with the management of chronic pain and comorbid psychiatric conditions are high and increasing, particularly in Western Europe where there is a progressive aging of the population [4]. The treatment of severe chronic pain often requires multidisciplinary rehabilitation in a hospital setting, while less severe cases may be managed with targeted interventions, such as physiotherapy or steroid administration, in primary healthcare [5].

Despite the possibility of contacting general practitioners or physiatrists, a significant percentage of people with chronic pain and related medical comorbidities consult firstly with physiotherapists to receive manual therapy [6]. In the light of the high prevalence of mood and anxiety disorders in subjects with chronic pain [7], it is important for physiotherapists to have the skills to identify mood and anxiety disorders in these patients [8]. Preliminary data show that physiotherapists have general positive attitudes towards mental health, although they often feel underprepared to work with patients affected by psychiatric disorders [9]. From a multidisciplinary perspective, specific training on these aspects would benefit physiotherapists in terms of proper referral of patients with psychiatric disorders to mental health professionals [10]. Of note, the presence of affective disorders can hamper adherence to rehabilitation programs, thus worsening patients’ prognosis [11].

The current literature shows that women are more likely to receive health care for musculoskeletal pain than men [12] and that gender role expectations can impact people’s perception and reporting of pain [13]. In addition, several authors identified a role of patients’ gender on decisions of healthcare providers [14]. On the contrary, few research studies have investigated the role of healthcare providers’ gender on processes of care. Preliminary data would indicate that having a female physician is associated with better communication with patients [15] and a higher quality of care [16]. With regard to lower back pain, two studies reported, respectively, that female physicians were more prone to prescribe pharmacological agents [17] as first choice and to refer patients to mental health professionals, despite evidence of organic pathology [18].

Although one role regarding physiotherapists’ gender has been recently hypothesized to affect clinical practice [19], research is very limited on this topic. Moreover, the sensitivity towards mental health issues could be different among physiotherapists depending on their gender [19]. The purpose of the present article is to identify eventual differences in the management of patients with persistent pain and comorbid anxiety/depression between male and female professionals.

## 2. Materials and Methods

### 2.1. Study Design

This is a cross-sectional study that had the objectives (1) to explore the educational needs of physiotherapists working in Italy and (2) to identify eventual differences between genders. A quantitative exploratory web-based cross-sectional survey was elaborated according to the Checklist for Reporting Results of Internet E-Survey (CHERRIES) guidelines [20] and the Strengthening the Reporting of Observational Studies in Epidemiology (STROBE) [21]. The survey was administered to physiotherapists in February 2022 (first two weeks).

### 2.2. Participants

The study included a nationwide sample of Italian physiotherapists with a degree in Physiotherapy (or equivalent legally recognized qualification) and enrolled in the TSRM-PSRT (Medical Radiology Health Technicians and Technical Rehabilitation Health Professions) professional register. The participants were identified by the AIFi (Italian Association of Physiotherapists) mailing list and its specialist groups including GTM (Manual Therapy and Musculoskeletal physiotherapy group) or in Facebook Groups for Physiotherapists. AIFi is the reference national scientific society for physiotherapists, while GTM is the Italian representative group of IFOMPT (International Federation of Orthopaedic Manipulative Physical Therapists). The target population consisted of 1146 colleagues. Among the established target population, we included those physiotherapists who: (a) had an e-mail account, (b) had a complete understanding of Italian language and (c) gave informed consent for study participation.

### 2.3. Questionnaire Development

An ad hoc questionnaire was developed for the objectives of the study. The initial list included 40 questions that were independently elaborated by one psychiatrist (MB) and one physiotherapist (MC). The questionnaire was revised by the members of the local Ethics Committee and a final survey with 35 questions was approved (see Table A1 for the corresponding questions to the investigated variables).

The final version of the questionnaire was divided into 3 sections (A, B and C); in section A, the socio-demographic variables were investigated by closed questions (e.g., gender and educational level); in section B, knowledge about depression/anxiety and attitudes towards the management of patients with pain and comorbid psychiatric conditions was investigated by closed questions and open questions (rating on a scale from 0 to 100); in section C, information about the desire and the need of psychiatric training for physiotherapists was collected.

Globally, information about the following variables was collected: age, gender, years of work experience, academic degree qualification, work settings (including location, e.g., urban area), main area of physiotherapy interest (e.g., orthopaedics or others), knowledge about medical comorbidities associated with chronic pain, awareness of the possible comorbidity of anxiety and depressive disorders in subjects affected by chronic pain, management of a patient affected by anxiety or depressive disorders, type of interaction with patients suffering from affective disorders, type of interaction with patients with persistent pain and psychiatric comorbidities (anxiety and depressive disorders), skills to identify the appropriate healthcare professional to refer the patients to in case of medical or psychiatric comorbidity, beliefs about adherence to treatments in subjects affected by anxiety and depressive disorders, beliefs about the beneficial effects of pharmacotherapy or psychotherapy for patients suffering from anxiety and depressive disorders, knowledge about side effects of psychopharmacotherapy, impact of psychopharmacotherapy on motor functions, level of education in psychiatry, awareness of the importance of recognition of mood and anxiety symptoms by the physiotherapist, the use of tools including rating scales to assess the presence or severity of psychiatric symptoms, past training about the screening of psychiatric symptoms, needs about further education in psychiatry.

### 2.4. Data Collection Procedures

The GoogleDoc online survey software (updated 2016 version, Upstartle, Portola Valley, CA, USA) was used to administer the questionnaire. After having obtained permission by AIFi and GTM, all mailing list subscribers were contacted by an e-mail containing the link to the survey and a brief note outlining the aim of the study, data handling (pseudo-anonymity), informed consent and privacy statement, invitation to complete the survey, presentation of the study and the authors. The respondents provided their consent to participate by clicking on the survey link.

Participation was voluntary and no incentives were offered to participants; it was possible to quit the questionnaire at any time. Participants were able to review or change the responses using a back button before submitting their answers.

Data were downloaded and stored in an encrypted computer and only the authors had access to the information during all stages of the study. Participants were ensured that their identities would not be disclosed by the investigators. All data were de-identified to maintain confidentiality and data protection.

### 2.5. Statistical Analysis

The sample size calculation was carried out as follows; given that a difference of at least 5 points (standard deviation: 15) was expected on the item “importance for physiotherapists to be trained in the recognition of anxiety and depressive symptoms” (on a scale from 0 = no importance to 100 = absolute importance) in males versus females and that a *p* value = 0.05 was considered statistically significant, for a power of 80%, a sample of at least 280 questionnaires (140 for each group) was calculated as reliable [19]. Descriptive analyses on the total sample were performed. Unpaired-sample *t* tests (for quantitative variables) and χ^2^ tests (for qualitative variables and with calculation of odds ratios (OR) when applicable) were performed to compare the groups identified by gender (none of the people interviewed declared a neutral gender). A binary logistic regression model (enter method) was then performed with factors that were significant in the univariate analyses as independent variables and gender as dependent one. The goodness of the models was verified by the Omnibus and Hosmer–Lemeshow tests.

The level of statistical significance was set at *p* ≤ 0.05 and the programme SPSS (version 27) was used to perform statistical analyses.

**Figure 1:**
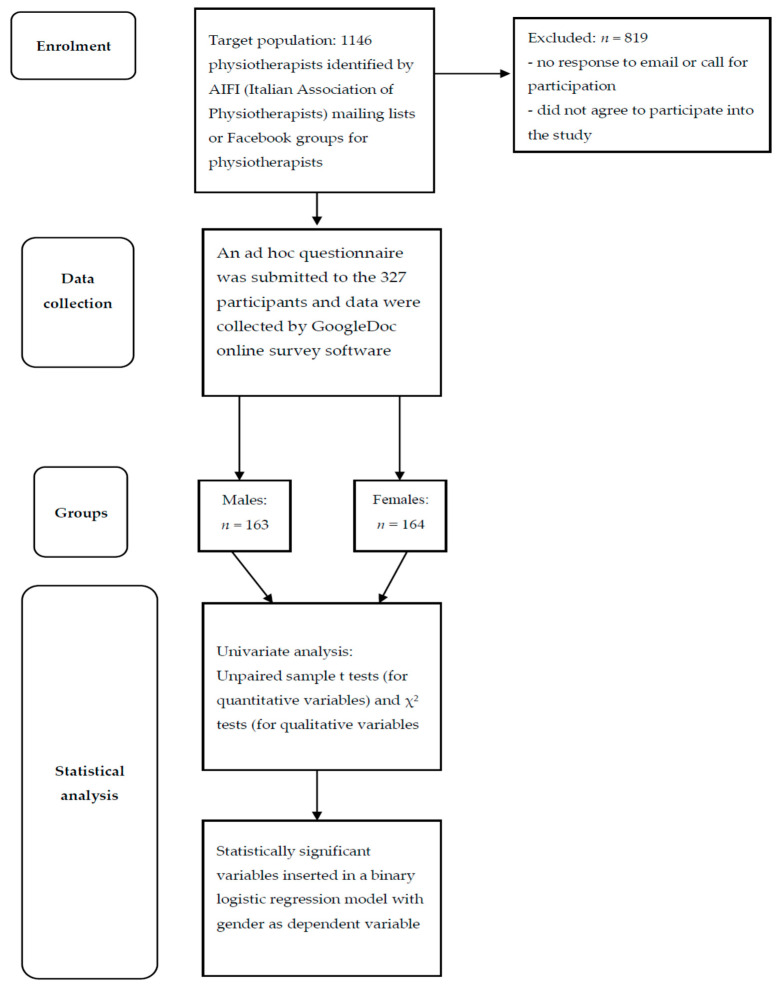
Flow diagram of the study.

Figure 1 summarizes the methodology used in the study.

## 3. Results

The number of respondents in the first two weeks of questionnaire diffusion was 327 (almost 30% of target population). In total, 49.8% of the respondents were men (*n* = 163) and 50.2% were women (*n* = 164) with an age between 23 and 68 years (mean age: 40.22 ± 10.04). Most physiotherapists had a 3-year degree (64.2%) without a further specialization (e.g., Masters). Most participants (34.6%) were experienced physiotherapists, doing this profession for 10 to 20 years, and 62.1% of respondents practiced in a private institution (i.e., private practice, private clinic). Orthopaedic rehabilitation was declared as the main area of interest by most of the participants (52.5%). Most of the respondents indicated a cognitive behavioural psychotherapist as the most appropriate mental health professional to refer patients with chronic pain and anxiety/depressive symptoms to (42.8%). Descriptive analyses of the total sample are reported in Table 1.

Female physiotherapists exhibited a higher level of confidence than male physiotherapists in administering continued physiotherapy for patients affected by Generalized Anxiety Disorder (GAD) (t = 2.46, *p* = 0.01), as well as for individuals who had previously engaged with a mental health professional (t = 2.79, *p* = 0.01). Furthermore, female physiotherapists versus male ones believed that pharmacotherapy was less associated with motor side effects (t = 2.90, *p* < 0.01) and more frequently recognized the importance of training to identify affective disorders (t = 2.65, *p* = 0.01) and the need for more education in mental health (t = 2.85, *p* = 0.01). In addition, female professionals worked less frequently as freelancers (χ^2^ = 29.46, *p* < 0.01, odds ratio—OR: 0.28 [confidence interval—CI: 0.17–0.44]) and with patients affected by musculoskeletal disorders (χ^2^ = 18.71, *p* < 0.01, OR: 0.38, CI: 0.24–0.59), feel more comfortable with patients affected by affective disorders (χ^2^ = 16.28, *p* < 0.01), but less comfortable in case of patients with affective disorders and concomitant pain (χ^2^ = 20.25, *p* < 0.01). The results of comparisons between genders are summarized in Table 1.

The binary logistic regression model with gender (female/male) as a dependent variable was reliable, allowing for a correct classification of 67.9% of the cases (Hosmer and Lemeshow Test: χ^2^ = 7.45, *p* = 0.49). The model was overall significant (Omnibus test: χ^2^ = 67.44, *p* < 0.01). In addition, no collinearity was identified between the predictors of the binary logistic regression model (variance inflation factor—VIF < 5). This analysis confirmed that female professionals (compared to male ones) were less likely to work freelance in private institutions (*p* = 0.015) and were more confident in the prosecution of physiotherapy by patients with GAD (*p* = 0.05) (Table 2).

## 4. Discussion

The results of the present article identified several gender differences in the approach of physiotherapists to patients with chronic pain and comorbid affective disorders, with an impact on the management of these subjects. One aspect that should be emphasized is that physiotherapists consider the cognitive behavioural psychotherapist as the main figure for referrals of patients with affective disorders. This aspect can be explained by the fact that physiotherapists prevalently manage patients with mild or moderate anxiety/depressive disorders, but it is also an indicator of the stigma associated with psychiatric treatment [22].

First of all, female professionals were more confident in the prosecution of therapy by patients affected by GAD, as also confirmed by the regression model. Some authors demonstrated that the level of confidence in patients’ treatment adherence by health professionals has a direct effect on prescription patterns, especially in case of chronic conditions [23]. Furthermore, an optimal communication between physiotherapists and patients with complete information about treatment can improve prescription compliance as a result of a perceived mutual trust [24]. It is also important to highlight that patients affected by GAD can be particularly prone to receive mechanic treatment or to perform exercises at home because fear of medications and related side effects is a frequent symptom of this condition [25]. Gender differences on this aspect can also be interpreted as the presence of a more positive attitude towards patients suffering from mental conditions in female versus male healthcare professionals [26].

Second, female physiotherapists (compared to males) were more confident in the receival of physiotherapy in patients who had been seen by a mental health professional, perhaps also as a result of more confidence in the tolerability of pharmacotherapy, especially regarding motor side effects. This finding could have been influenced by the fact that in our sample female professionals worked more frequently in places of care than as freelancers, so they could benefit from direct cooperation with other health specialists and be more confident in multidisciplinary cooperation for the management of patients with chronic pain. Of note, the current literature indicates that multidisciplinary biopsychosocial interventions are very effective in subjects affected by chronic lower back pain [27]. This positive attitude by female physiotherapists could have been enhanced by the fact that in our sample, women compared to men more frequently had an area of interest other than musculoskeletal conditions.

Finally, female professionals recognized more than males the importance of identifying affective disorders for better management of patients with chronic pain. In addition, they reported (more than their counterparts) the need for further training on psychiatry. The application of a biopsychosocial model in the management of chronic pain is crucial because increasing evidence indicates that the presence of depressive and anxiety disorders are factors associated with the persistence of physical pathologies [28,29]. A recent article highlighted that physiotherapists could provide mental health interventions autonomously, such as graded exercise or graded activity, but that insufficient knowledge is one the most prominent barriers in applying these types of interventions [30]. Mental health training among physical therapists should therefore be promoted to overcome these barriers. Male professionals represent the target of educational events finalized to raise awareness about the importance of patients’ psychological well-being in obtaining amelioration of chronic pain. Our data would indicate that male professionals (compared to female ones) are hesitant to abandon a traditional model of physiotherapy in favour of intervention strategies that take into account emotional aspects [30].

The study has some limitations: (1) the subjectivity of self-rating, (2) the lack of validation of the administered interview, (3) the different provenience of the respondents who have a dissimilar attitude to psychiatric disorders according to the organization of the local health system.

## 5. Conclusions

In conclusion, female physiotherapists (compared their counterparts) appear to have a more positive attitude towards patients with chronic pain and psychiatric comorbidity, and they are more aware of the benefits of mental health training to improve the prognosis of these patients. Psychoeducational initiatives could be implemented to diminish stereotypes or the stigma associated with mental illness, particularly for male physiotherapists. This is particularly relevant as different attitudes towards mental illness between male and female medical students have been reported early, with this gap narrowing after adequate training in psychiatry [31]. In this sense, a biopsychosocial approach to chronic pain could improve the prognosis of patients with chronic pain, as well having as a multidisciplinary contribution by overcoming old treatment schemes [27]. Further studies, collecting data from different countries to assess the effect of local cultural and psychosocial factors, are needed to confirm the results of the present study.

## Figures and Tables

**Table 1:** Summary of the results of the total sample and of the two groups identified by gender.

Variable		Total Sample *n* = 327	Males *n* = 163	Females *n* = 164	*p*
Age		40.22 (±10.04)	39.17 (±9.97)	41.26 (±10.03)	0.06
Years of work experience	*<5 years*	33 (10.1%)	17 (10.4%)	16 (9.8%)	0.08
	*from 5 to 10 years*	81 (24.8%)	50 (30.7%)	31 (18.9%)	
	*from 10 to 20 years*	113 (34.6%)	53 (32.5%)	60 (36.6%)	
	*>20 years*	100 (30.5%)	43 (26.4%)	57 (34.7%)	
Italian area Missing = 2	*Northwest*	109 (33.5%)	50 (31.1%)	59 (36.0%)	0.14
	*Northeast*	119 (36.6%)	55 (34.2%)	64 (39.0%)	
	*Central*	60 (18.5%)	37 (22.9%)	23 (14.0%)	
	*South*	25 (7.7%)	15 (9.3%)	10 (6.1%)	
	*Islands*	12 (3.7%)	4 (2.5%)	8 (4.9%)	
Work setting	*Private*	124 (37.9%)	38 (23.3%)	86 (52.4%)	**<0.01**
	*Others*	203 (62.1%)	125 (76.7%)	78 (47.6%)	
Work area (size population) Missing = 1	*>500,000*	55 (16.6%)	31(19.0%)	23 (14.1%)	0.65
	*>100,000 < 500,000*	74 (22.7%)	37 (22.7%)	37 (22.7%)	
	*15,000–100,000*	84 (25.7%)	39 (23.9%)	45 (27.6%)	
	*<15,000*	114 (35.0%)	56 (34.4%)	58 (35.6%)	
Main area of physiotherapy interest Missing = 1	*Musculoskeletal disorders*	155 (47.5%)	58 (35.6%)	97 (59.5%)	**<0.01**
	*Others*	171 (52.5%)	105 (64.4%)	66 (40.5%)	
Medical comorbidity that is considered to be more associated with chronic pain Missing = 3	*Diabetes*	124 (38.3%)	55 (33.7%)	69 (42.9%)	0.15
	*Cardiovascular diseases*	78 (24.1%)	44 (27.0%)	34 (21.1%)	
	*Respiratory diseases*	19 (5.8%)	7 (4.3%)	12 (7.5%)	
	*Mental disorders*	103 (31.8%)	57 (35.0%)	46 (28.5%)	
Hypothesized percentage of patients with concomitant chronic pain and GAD Missing = 10		61.27 (±23.31)	59.92 (±22.78)	62.62 (±23.83)	0.30
Hypothesized percentage of treatment withdrawal in patients with concomitant chronic pain and GAD Missing = 13		40.36 (±24.30)	43.73 (±24.17)	37.03 (±24.04)	**0.01**
Hypothesized percentage of patients with concomitant chronic pain and depressive disorders Missing = 11		57.26 (±26.34)	58.03 (±25.40)	56.51 (±27.28)	0.61
Hypothesized percentage of treatment withdrawal in patients with concomitant chronic pain and depressive disorders Missing = 14		43.75 (±26.43)	46.39 (±26.32)	41.13 (±26.36)	0.08
Referred percentage of patients with persistent pain treated by the physiotherapists Missing = 6		33.47 (±25.34)	34.07 (±25.29)	32.88 (±25.45)	0.67
Percentage of patients with chronic pain who are believed to accept the indication to consult a mental health professional Missing = 11		30.22 (±21.81)	31.90 (±21.22)	28.51 (±22.34)	0.17
Percentage of patients with chronic pain who are believed to withdraw physiotherapy after visit with a mental health professional Missing = 14		24.84 (±22.56)	28.35 (±23.29)	21.30 (±21.29)	**0.01**
Percentage of patients with chronic pain and comorbid mood/anxiety disorders who are believed to benefit from pharmacotherapy Missing = 23		42.81 (±25.14)	45.07 (±25.20)	40.55 (±24.95)	0.12
Percentage of patients with chronic pain and comorbid mood/anxiety disorders who are believed to benefit from psychotherapy Missing = 16		76.39 (±22.47)	75.45 (±19.52)	77.33 (±25.08)	0.46
Knowledge of side effects of pharmacotherapy * Missing = 7		37.15 (±28.28)	38.68 (±28.79)	35.63 (±27.76)	0.34
Degree of agreement with the statement: “psychopharmacological therapy negatively affects motor performance” * Missing = 17		46.77 (±28.91)	51.45 (±29.30)	42.03 (±27.82)	**<0.01**
Participation in psychiatry training events Missing = 2	*No*	250 (76.9%)	124 (76.5%)	126 (77.3%)	0.87
	*Yes*	75 (23.1%)	38 (23.5%)	37 (22.7%)	
Importance for physiotherapists to be trained in the recognition of anxiety and depressive symptoms * Missing = 3		87.55 (±18.13)	84.91 (±19.26)	90.20 (±16.57)	**0.01**
Use of rating scales to assess anxiety and depressive symptoms in patients with chronic pain Missing = 2	*No*	272 (83.7%)	131 (80.9%)	141 (86.5%)	0.17
	*Yes*	53 (16.3%)	31 (19.1%)	22 (13.5%)	
Observation of the administration of rating scales for anxiety and depressive symptoms Missing = 2	*No*	232 (71.4%)	120 (74.1%)	112 (68.7%)	0.29
	*Yes*	93 (28.6%)	42 (25.9%)	51 (31.3%)	
Attendance of training courses to administer psychiatric rating scales Missing = 3	*No*	298 (92.0%)	149 (92.0%)	149 (92.0%)	1.00
	*Yes*	26 (8.0%)	13 (8.0%)	13 (8.0%)	
Utility of more mental health training for the physiotherapists *		81.79 (±22.03)	78.33 (±23.55)	85.26 (±19.88)	**0.01**
Ability to interact with patients affected by mood and anxiety disorders	*Perfectly comfortable*	37 (11.3%)	26 (16.0%)	11 (6.7%)	**<0.01**
	*Usually comfortable*	158 (48.3%)	76 (46.6%)	82 (50.0%)	
	*Neutral*	22 (6.7%)	12 (7.4%)	10 (6.1%)	
	*Sometimes uncomfortable*	86 (26.3%)	32 (19.6%)	54 (32.9%)	
	*Often uncomfortable*	24 (7.4%)	17 (10.4%)	7 (4.3%)	
Ability to interact with patients with chronic pain and comorbid mood or anxiety disorders	*Perfectly comfortable*	33 (10.1%)	24 (14.7%)	9 (5.5%)	**<0.01**
	*Usually comfortable*	141 (43.1%)	68 (41.7%)	73 (44.5%)	
	*Neutral*	36 (11.1%)	23 (14.2%)	13 (7.9%)	
	*Sometimes uncomfortable*	92 (28.1%)	32 (19.6%)	60 (36.6%)	
	*Often uncomfortable*	25 (7.6%)	16 (9.8%)	9 (5.5%)	
Utility of mental health screening in patients suffering from chronic pain Missing = 2	*No, I am just interested in my practice*	1 (0.3%)	1 (0.6%)	0 (0.0%)	0.55
	*No, it is not cost effective*	11 (3.4%)	7 (4.3%)	4 (2.5%)	
	*Yes, it should be done for selected patients*	230 (70.8%)	115 (71.0%)	115 (70.5%)	
	*Yes, it should be done for all patients*	83 (25.5%)	39 (24.1%)	44 (27.0%)	
Presence of a trusted professional to refer patients to after screening for anxiety or depressive symptoms	*No*	76 (46.6%)	87 (53.0%)	163 (49.8%)	0.25
	*Yes*	87 (53.4%)	77 (47.0%)	164 (50.2%)	

Legend: * on a scale ranging from 0 = totally no to 100 = totally yes. GAD: Generalized Anxiety Disorder. *p*: *p* values. In bold statistically significant *p* from unpaired-sample *t* tests (quantitative variables) and χ^2^ tests (qualitative variables).

**Table 2:** Summary of the results of logistic regression model.

Variables	B	SE	*p*	OR	95% CI
Hypothesized percentage of treatment withdrawal in patients with concomitant chronic pain and GAD	−0.011	0.006	**0.050**	0.989	0.977–0.999
Percentage of patients with chronic pain who are believed to withdraw physiotherapy after visit with a mental health professional	−0.010	0.007	0.128	0.990	0.977–1.003
Degree of agreement with the statement: “psychopharmacological therapy negatively affects motor performance” *	−0.009	0.005	0.085	0.991	0.981–1.001
Importance for physiotherapists to be trained in the recognition of anxiety and depressive symptoms *	0.017	0.009	0.080	1.017	0.998–1.036
Utility of more mental health training for the physiotherapists *	0.009	0.008	0.268	1.009	0.993–1.024
Work setting (private versus others)	−0.775	0.319	**0.015**	0.461	0.246–0.860
Musculoskeletal disorders as the main area of interest (Yes versus No)	−0.431	0.304	0.157	0.650	0.358–1.180
Ability to interact with patients affected by mood and anxiety disorders	NA	NA	0.442	NA	NA
Ability to interact with patients with chronic pain and comorbid mood or anxiety disorders	NA	NA	0.076	NA	NA

Legend: * on a scale ranging from 0 = totally no to 100 = totally yes. B: regression coefficient; CI: confidence interval; GAD: Generalized Anxiety Disorder; NA: not applicable; OR: odds ratio; SE: standard error. In bold, statistically significant *p* values (*p* ≤ 0.05).

## Data Availability

The data used to support the findings of this study are available from the corresponding author upon reasonable request.

## Appendix A

**Table A1:** Observed variable and related questions.

Variable	Question
Hypothesized percentage of patients with concomitant chronic pain and GAD	What is the PERCENTAGE of patients with persistent pain who may have an attitude of extreme concern and a general pessimistic view about their future along with sleep disturbances and muscle tension?
Hypothesized percentage of treatment withdrawal in patients with concomitant chronic pain and GAD	What is the PERCENTAGE of patients, with the symptoms of the previous question, that you think can early withdraw an effective therapeutic program?
Hypothesized percentage of patients with concomitant chronic pain and depressive disorders	What is the PERCENTAGE of patients with persistent pain who may have depressed mood for at least two weeks possibly together with a general reduction in interest, difficulties of concentration, sleep disturbances, decreased appetite, reduced energy, feelings of guilt and social dysfunction?
Hypothesized percentage of treatment withdrawal in patients with concomitant chronic pain and depressive disorders	What is the PERCENTAGE of patients, with the symptoms of the previous question that you think can early withdraw an effective therapeutic program?
Referred percentage of patients with persistent pain treated by the physiotherapists	What is the PERCENTAGE of patients with persistent pain episodes who you treat?
Utility of mental health screening in patients suffering from chronic pain	Do you think that mental health screening can be useful to improve prognosis and to reduce healthcare costs in patients suffering from chronic pain?
Ability to interact with patients affected by mood and anxiety disorders	How much do you feel confident in interact with patients affected by anxiety disorders or depression?
Ability to interact with patients with chronic pain and comorbid mood or anxiety disorders	How much do you feel confident to interact with patients affected by chronic pain and comorbid anxiety or depressive disorders?
Presence of a trusted professional to refer patients to after screening for anxiety or depressive	Do you have a trusted professional to refer patients after screening for anxiety disorders and depression?
Percentage of patients with chronic pain who are believed to accept the indication to consult a mental health professional	What is the expected PERCENTAGE of patients that you think will accept the referral to a mental health professional?
Percentage of patients with chronic pain who are believed to withdraw physiotherapy after visit with a mental health professional	What is the expected PERCENTAGE of patients that you think will drop out of physiotherapy after consulting a mental health professional?
Percentage of patients with chronic pain and comorbid mood/anxiety disorders who are believed to benefit from pharmacotherapy	What is the PERCENTAGE of patients with symptoms of anxiety and/or depression who can benefit from a pharmacological approach in your opinion?
Percentage of patients with chronic pain and comorbid mood/anxiety disorders who are believed to benefit from psychotherapy	What is PERCENTAGE of patients with symptoms of anxiety and/or depression who can benefit from a psychotherapeutic approach in your opinion?
Knowledge of side effects of pharmacotherapy	How much do you know about the side effects of the medications available to treat symptoms of anxiety and depression? Rate on a scale from 0 (no knowledge) to 100 (full knowledge).
Degree of agreement with the statement: “psychopharmacological therapy negatively affects motor performance”	Could you express your degree of agreement regarding this statement: “psychopharmacological therapy negatively affects motor performance”. Rate on a scale from 0 (no agreement) to 100 (complete agreement).
Participation in psychiatry training events	Did you attend psychiatric educational or training courses?
Importance for physiotherapists to be trained in the recognition of anxiety and depressive symptoms	How much the identification of anxiety and depression symptoms is relevant for a physiotherapist? Rate on a scale from 0 (totally no) to 100 (totally yes).
Use of rating scales to assess anxiety and depressive symptoms in patients with chronic pain	Did you ever screen your patients with chronic pain by rating scales assessing depression and anxiety?
Observation of the administration of rating scales for anxiety and depressive symptoms	Have you never assisted to the administration of rating scales to assess the presence of anxiety and depression?
Attendance of training courses to administer psychiatric rating scales	Have you never attended training courses to administer psychiatric rating scales?
Utility of more mental health training for the physiotherapists	How much can a training on mental health be useful for your profession? Rate on a scale from 0 (totally no) to 100 (totally yes).

Legend: GAD: Generalized Anxiety Disorder.
